# Impact of proton PBS machine operating parameters on the effectiveness of layer rescanning for interplay effect mitigation in lung SBRT treatment

**DOI:** 10.1002/acm2.14342

**Published:** 2024-04-08

**Authors:** Xiaoying Liang, Chunbo Liu, Jiajian Shen, Stella Flampouri, Justin C. Park, Bo Lu, Sridhar Yaddanapudi, Jun Tan, Keith M. Furutani, Chris J. Beltran

**Affiliations:** ^1^ Department of Radiation Oncology Mayo Clinic Jacksonville Florida USA; ^2^ Department of Radiation Oncology The First Affiliated Hospital of Zhengzhou University Zhengzhou China; ^3^ Department of Radiation Oncology Mayo Clinic Phoenix Arizona USA; ^4^ Department of Radiation Oncology Winship Cancer Institute Emory University Atlanta USA

**Keywords:** interplay effect, layer rescanning, lung SBRT, machine operation parameters, proton PBS

## Abstract

**Background:**

Rescanning is a common technique used in proton pencil beam scanning to mitigate the interplay effect. Advances in machine operating parameters across different generations of particle therapy systems have led to improvements in beam delivery time (BDT). However, the potential impact of these improvements on the effectiveness of rescanning remains an underexplored area in the existing research.

**Methods:**

We systematically investigated the impact of proton machine operating parameters on the effectiveness of layer rescanning in mitigating interplay effect during lung SBRT treatment, using the CIRS phantom. Focused on the Hitachi synchrotron particle therapy system, we explored machine operating parameters from our institution's current (2015) and upcoming systems (2025A and 2025B). Accumulated dynamic 4D dose were reconstructed to assess the interplay effect and layer rescanning effectiveness.

**Results:**

Achieving target coverage and dose homogeneity within 2% deviation required 6, 6, and 20 times layer rescanning for the 2015, 2025A, and 2025B machine parameters, respectively. Beyond this point, further increasing the number of layer rescanning did not further improve the dose distribution. BDTs without rescanning were 50.4, 24.4, and 11.4 s for 2015, 2025A, and 2025B, respectively. However, after incorporating proper number of layer rescanning (six for 2015 and 2025A, 20 for 2025B), BDTs increased to 67.0, 39.6, and 42.3 s for 2015, 2025A, and 2025B machine parameters. Our data also demonstrated the potential problem of false negative and false positive if the randomness of the respiratory phase at which the beam is initiated is not considered in the evaluation of interplay effect.

**Conclusion:**

The effectiveness of layer rescanning for mitigating interplay effect is affected by machine operating parameters. Therefore, past clinical experiences may not be applicable to modern machines.

## 1 INTRODUCTION

Stereotactic Body Radiation Therapy (SBRT)^1^ has demonstrated its effectiveness for treating early‐stage non‐small cell lung cancer (NSCLC).^2^, ^3^, ^4^ In recent years, proton therapy, an evolving radiotherapy modality, has gained increasing interest for lung SBRT treatment.^5^, ^6^, ^7^ Studies have shown the superiority of proton SBRT over photon SBRT in providing conformal dose distributions and sparing doses to organs at risk (OAR).^8^, ^9^, ^10^, ^11^ However, when treating a moving target, the interplay effect in proton pencil beam scanning (PBS) remains a significant challenge that requires careful consideration and mitigation as reviewed in AAPM task TG‐290^12^ and references therein. The interplay effect arises from the dynamic nature of both the scanned proton beam and the movement of the tumor, potentially leading to substantial cold and hot spots in the delivered dose and significantly degrading the dose distribution.^13^, ^14^, ^15^, ^16^, ^17^

As detailed in AAPM task group TG290,^12^ the methods for interplay effect mitigation includes rescanning, breath‐hold, gating, and tumor tracking. Among these, rescanning is a relatively straightforward way to deal with the interplay effect, as such it stands out as a widely adopted method in clinical practice.^18^, ^19^, ^20^, ^21^, ^22^, ^23^ Rescanning involves delivering spots multiple times, essentially performing statistical averaging of the spot delivery across breathing phases. Consequently, the time of rescanning plays an important role on its efficacy for interplay effect mitigation. In the last decade, notable technological progress has led to advanced machine operating parameters, resulting in a substantial improvement in beam delivery time (BDT).^24^ As a result, the time structure of the beam delivery, and consequently the time structure of rescanning, can vary significantly among different generations of machines. This variability might impact the effectiveness of rescanning in mitigating the interplay effect. Therefore, the clinical experience gained in the past may not be applicable to the latest machines. However, reports on this important topic are lacking.

This study aims to address this knowledge gap by investigating the impact of machine operating parameters on the effectiveness of rescanning for interplay effect mitigation in lung SBRT treatment. Specifically, our focus is on the Hitachi synchrotron particle therapy system, examining machine operating parameter sets, including those currently in operation at our institution since 2015 and those of our upcoming particle therapy system. Additionally, we explored the implications of layer rescanning on the total BDT to provide valuable insights for both precision dose delivery and efficiency proton treatment in lung SBRT.

## 2 MATERIAL AND METHODS

### 2.1 Machine operating parameters

Three sets of machine operating parameters from Hitachi synchrotron particle therapy system with multi‐energy extraction^25^ were included in the study (Table 1). One set of parameters is from the system operational since 2015 at our institution (referred to as 2015), while the other two sets are from our upcoming system scheduled for installation in 2025 and are referred as 2025A and 2025B.

**TABLE 1:** Machine operating parameters.

Parameters^a^	2015	2025A	2025B
Scanning speed in [x,y] direction for 230 MeV protons (m/s)^b^	[6,10]	[8,20]	[40,120]
Scanning magnet preparation and verification Time (ms)	1.9	1.9	1.1
Spill change time (s)	2	2	2
Maximum MU per spill	20	30	60
Number of deliverable MEE layers per spill	4	16	unlimited
Recapture efficiency (%)	50	80	100
MEE layer switch time (s)	0.2	0.2	0.1
Beam current (MU/s)	8	30	40

Abbreviations: MEE, multiple energy extraction; MU, monitor unit.

^a^ All parameters in this table are energy independent except scanning speeds.

^b^ Only scanning speeds for the 230 MeV are listed. The scanning speeds for the other proton energies can be derived from the speeds of the max energy.^29^ ^.^

### 2.2 CT simulation and treatment planning

This study utilized the CIRS dynamic anthropomorphic thorax phantom (CIRS Inc., USA), which is engineered to simulate the typical human thorax. Constructed with materials that mimic lung, bone, and soft tissue, the CIRS phantom incorporates a 25‐mm soft tissue‐equivalent target situated on a movable lung‐equivalent rod within the lung‐equivalent section. We simulated a sinusoidal tumor motion with a 5‐s respiratory cycle and 10‐mm motion amplitudes. A four‐dimensional computed tomography (4DCT) scan was acquired using a Siemens CT (Siemens Healthineers, Munich, Germany). The 10‐phases and average CT images were transferred to MIM (MIM Software Inc., Beachwood, OH) for contouring. The gross tumor volume (GTV) on each phase and the internal gross tumor volume (IGTV) on the average CT were contoured. Subsequently, the CT images and the contours were transferred to Eclipse treatment planning system (TPS) V15.6 (Varian Medical Systems, Palo Alto, CA) for treatment planning. The planning was conducted on the average CT, with a prescribed dose of 50 Gy (RBE) in five fractions to IGTV. The particle therapy system is commissioned with proton energies ranged from 71.3 to 228.8 MeV. The spot sigma in air at isocenter ranges from 2 to 6 mm. In our system 1 MU corresponds to approximately 0.9 billion protons at 228.8 MeV and approximately 0.4 billion protons at 71.3 MeV.^26^ A single posterior‐anterior (PA) beam with approximately 5 mm (1 sigma in water) spot spacing was used for planning. The plan was robustly optimized on IGTV with setup uncertainties (5 mm) and range uncertainties (3.5%), parameters commonly utilized in proton lung cancer treatment planning.^27^

### 2.3 Layer rescanning

The rescanning technique under investigation in our study is scaled layer rescanning,^13^ where each spot in a single energy layer is rescanned a fixed number of times before proceeding to the next energy layer. In the remainder of the text, we use the term “layer rescanning” to refer to scaled layer rescanning.

The initial treatment plan was post‐processed to have each spot on each layer to be rescanned 4, 6, 8, 10, 12, 16, 20, and 24 times. In the rescanning plan with *n* times of layer rescanning, all spots in a layer were scanned with spot MUs scaled down to *1/n* of the original MUs, and this pattern of layer scanning was repeated *n* times to reach the desired MU:

$$\;MU_{i,\;\left(i+m\right),\text{…},\left(i+\left(n-1\right)m\right)}^{j,\;rescanning\;plan}=\;MU_{i}^{j,\;initial\;plan}/n$$

where $MU_{i}^{j}\;$ represent the MU of spot index of *i* at layer *j*. *m* is the total number of spots in layer *j*. The layer rescanning is achieved by equally distribute the initial plan's $MU_{i}^{j}$ to the spots with indices of i,(i+m),…,(i+(n−1)m) in layer *j* in the rescanning plan.

### 2.4 Interplay effect evaluation

The interplay effect and the effectiveness of its mitigation method were assessed by the accumulated dynamic 4D dose (D4DD). The D4DD incorporates the time‐dependent beam delivery sequence along with anatomical motion, and deformable accumulation of the dose to the reference respiratory phase.^28^

**FIGURE 1:**
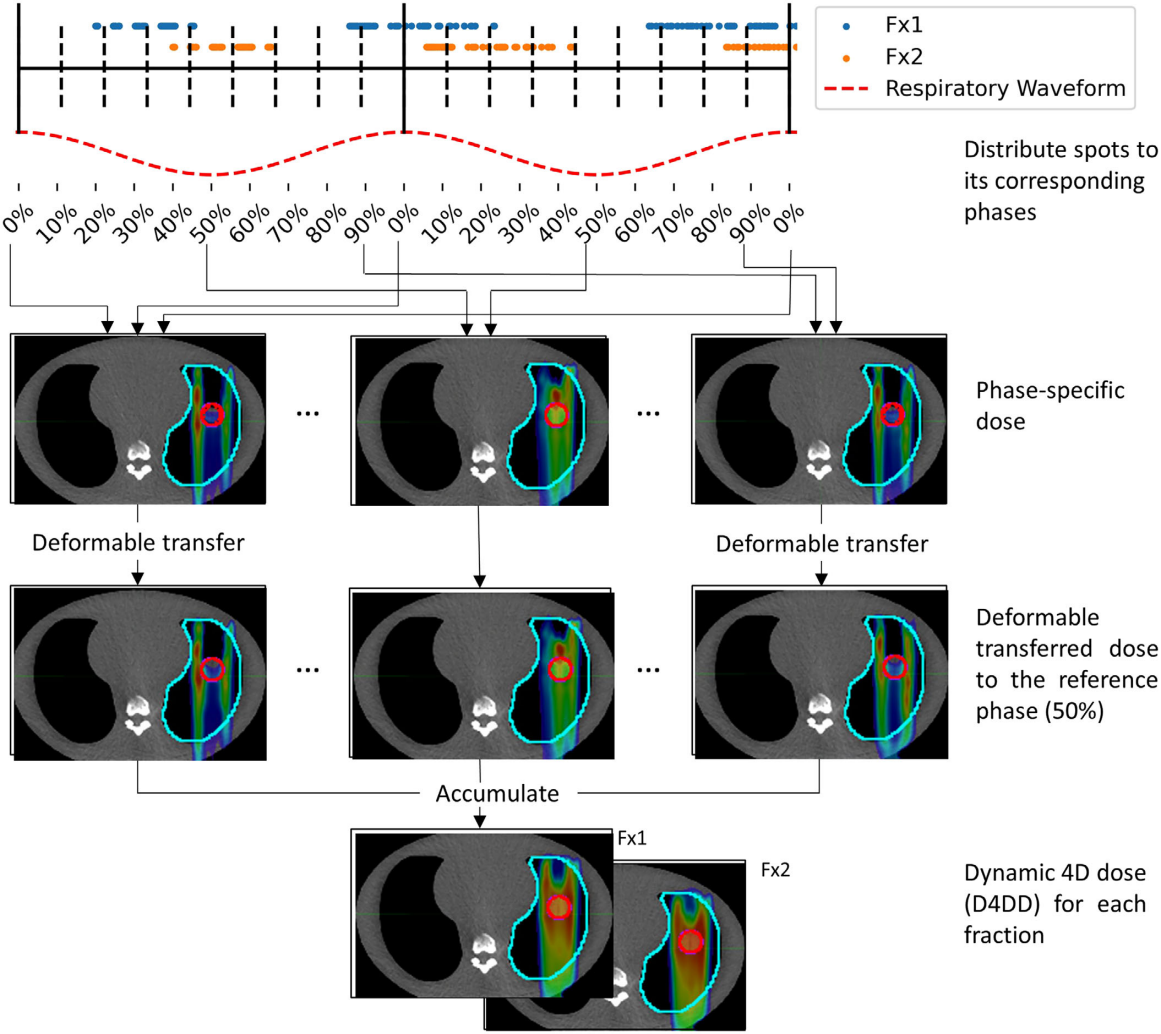
D4DD dose reconstruction workflow.

The workflow of the D4DD reconstruction is depicted in Figure 1. Firstly, using an in‐house developed beam delivery model, each spot's delivery time stamp was simulated based on the beam delivery sequence and machine operating parameters. The details of the beam delivery model and its validation can be found in our previous publication.^29^ Briefly, the beam delivery consists of energy switch time, spot switch time, and active beam‐on time. The starting phase assignment for the first spot was randomly placed in one of the 10 respiratory phases using a uniform distribution. Each subsequent spot was temporarily assigned to the corresponding respiratory phase based on the calculated time stamp and the initial spot assignment. Subsequently, the phase‐specific spots maps were imported into the Eclipse TPS. The phase‐specific dose was then computed on the corresponding phase CT. Thereafter, each phase's dose was transferred to MIM and deformably accumulated to the 50% phase.

This D4DD reconstruction process was repeated for each fraction. The D4DD from each fraction were then summed to obtain the accumulated D4DD. The accumulated D4DD to the GTV was compared to the IGTV dose in the reference plan. Target coverage was assessed using the dose received by 95% of the target volume (D95). The evaluation of hot and cold spots was assessed by the ratio of the dose received by 5% of the target to that received by 95% of the target (D5/D95).^23^ The mean dose and V_20GyRBE_ to the healthy ipsilateral lung (i.e., ipsilateral Lung—IGTV) was evaluated as well.

The BDT was evaluated for different number of layer scanning, to provide insights for both precision in dose distribution and treatment efficiency. Additionally, we evaluated fractional D4DD across the five fractions. As explained earlier, in each fraction, the initial spot was randomly placed in one of the 10 respiratory phases. Therefore, analyzing the fractional D4DD across the five fractions will provide insights into the sensitivity of the randomness in the initiation of beam delivery with respect to different respiratory phases.

## 3 RESULTS

### 3.1 Accumulated D4DD

**FIGURE 2:**
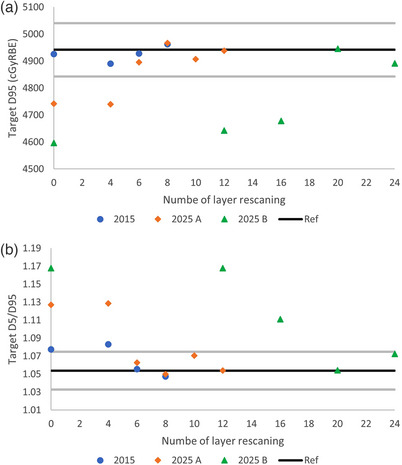
The accumulated D4DD on (a) GTV D95 and (b) GTV D5/D95 for the three sets of machine operating parameters, each with various numbers of layer rescanning. The black line represents the reference values on IGTV in the reference plan. The gray lines represent ±2% deviation from the reference values.

Figure 2(a) displays the accumulated D4DD GTV D95 and Figure 2(b) depicts the GTV D5/D95 ratio for different sets of machine operating parameters, each with various number of layer rescanning. The reference values as well as ±2% deviations from the reference plan are also presented for comparison.

In Figure 2, it is evident that when the rescanning technique is not employed, the interplay effect degrades the dose distribution with varying degrees of severity among different sets of machine operating parameters. The more advanced machine operating parameters resulted in a more pronounced under‐dosage to the target and increased hot and cold spots. For the 2015 machine parameters, although it achieved acceptable D95 coverage without the need for rescanning, the interplay effect caused hot spots and the ratio of D5/D95 deviated more than 2% from the reference plan.

To achieve the accumulated D4DD close to the planned one within 2% deviation, rescanning technique is required. The machine with the most advanced operating parameters required the highest number of layer rescanning. For the 2025B machine parameters, 20 times layer rescanning is required to achieve proximity to the planned target coverage and homogeneous dose distribution. In contrast, for the other two sets of machine parameters, six times layer rescanning is sufficient. It is also observed that beyond these points, keep increasing the number of layer rescanning does not yield further improvements in the dose distribution. Using the data from the 2025A machine as example, increasing the number of layer rescanning from 6 to 8, 10, and 12 does not result in a monotonic improvement in the dose distribution. This suggests that, for each setting and given case, identifying an optimal number of layer rescanning is beneficial for achieving efficient treatment while maintaining acceptable dose accuracy.

**FIGURE 3:**
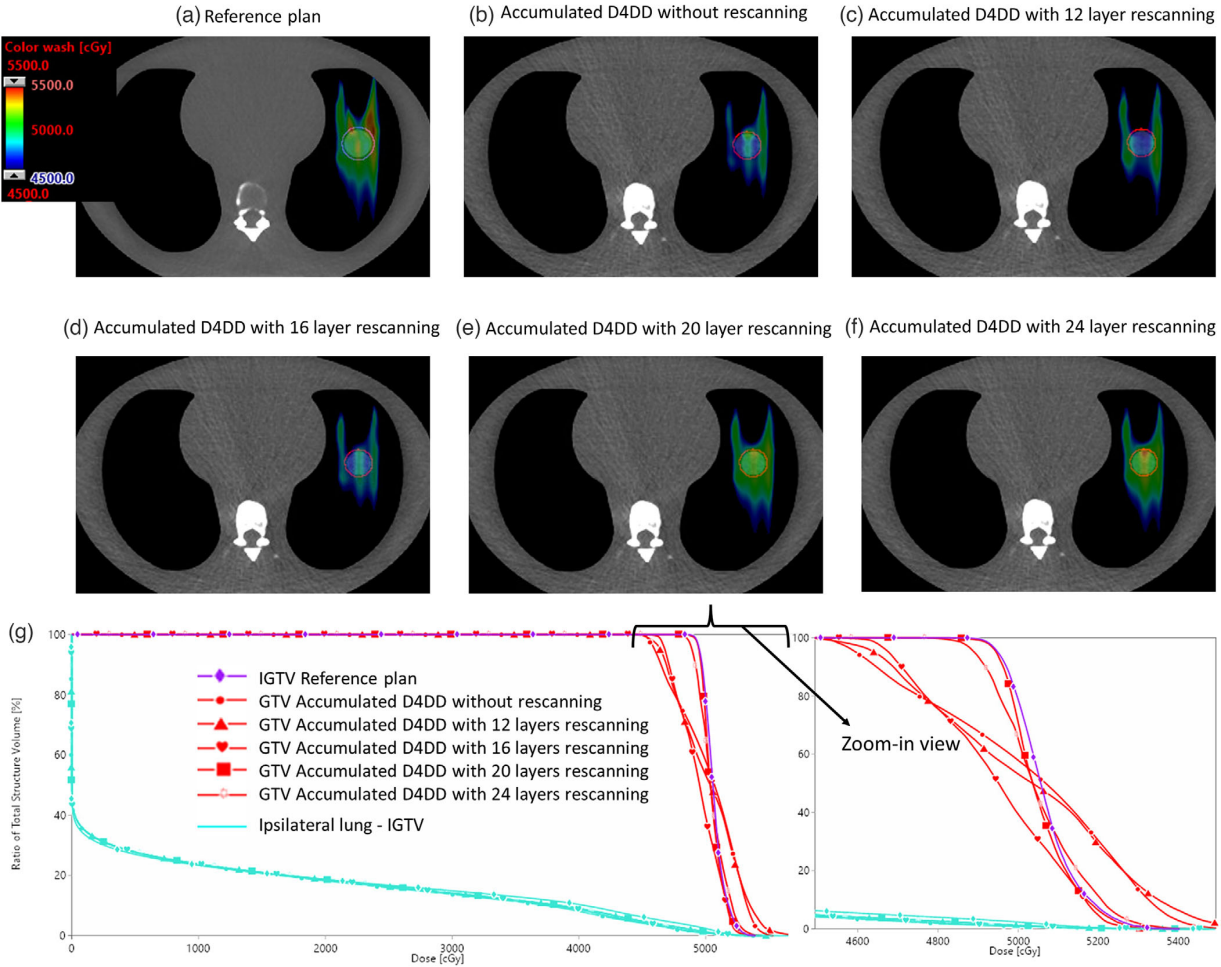
Color washed accumulated D4DD distribution for the 2025B machine parameters. (a) dose distribution of the reference plan, (a) without rescanning, (c) with 12 layer rescanning, (d) with 16 layer rescanning, (e) with 20 layer rescanning, and (f) with 24 layer rescanning. The DVH comparison is also presented (g).

Taking the 2025B machine parameters as an example, Figure 3 displays the color washed accumulated D4DD for 0, 12, 16, 20, and 24 times of layer rescanning. The dose distribution of the reference plan is included for comparison. The DVH comparison is also presented. Clear evidence of dose degradation, with underdosing of the target and significant hot and cold spots, was observed in accumulated D4DDs without the rescanning or with fewer than 20 times layer rescanning. When the number of layer rescanning equals or greater than 20, the dose distribution became closely matched the planned one. From the DVH plot in Figure 3, we also overserved that the interplay effect has minimal impact on the dose distribution to the (Ipsilateral lung—IGTV). In the reference plan, V20 and mean dose to this structure were 18.8% and 836 cGy (RBE), respectively. The accumulated D4DD showed V20 and mean dose of 18.6% and 829 cGy (RBE) without rescanning. With layer rescanning, these values ranged from 18.4% to 18.8% for V20 and from 826 cGy(RBE) to 837 cGy(RBE) for the mean dose.

### 3.2 Beam delivery time

**FIGURE 4:**
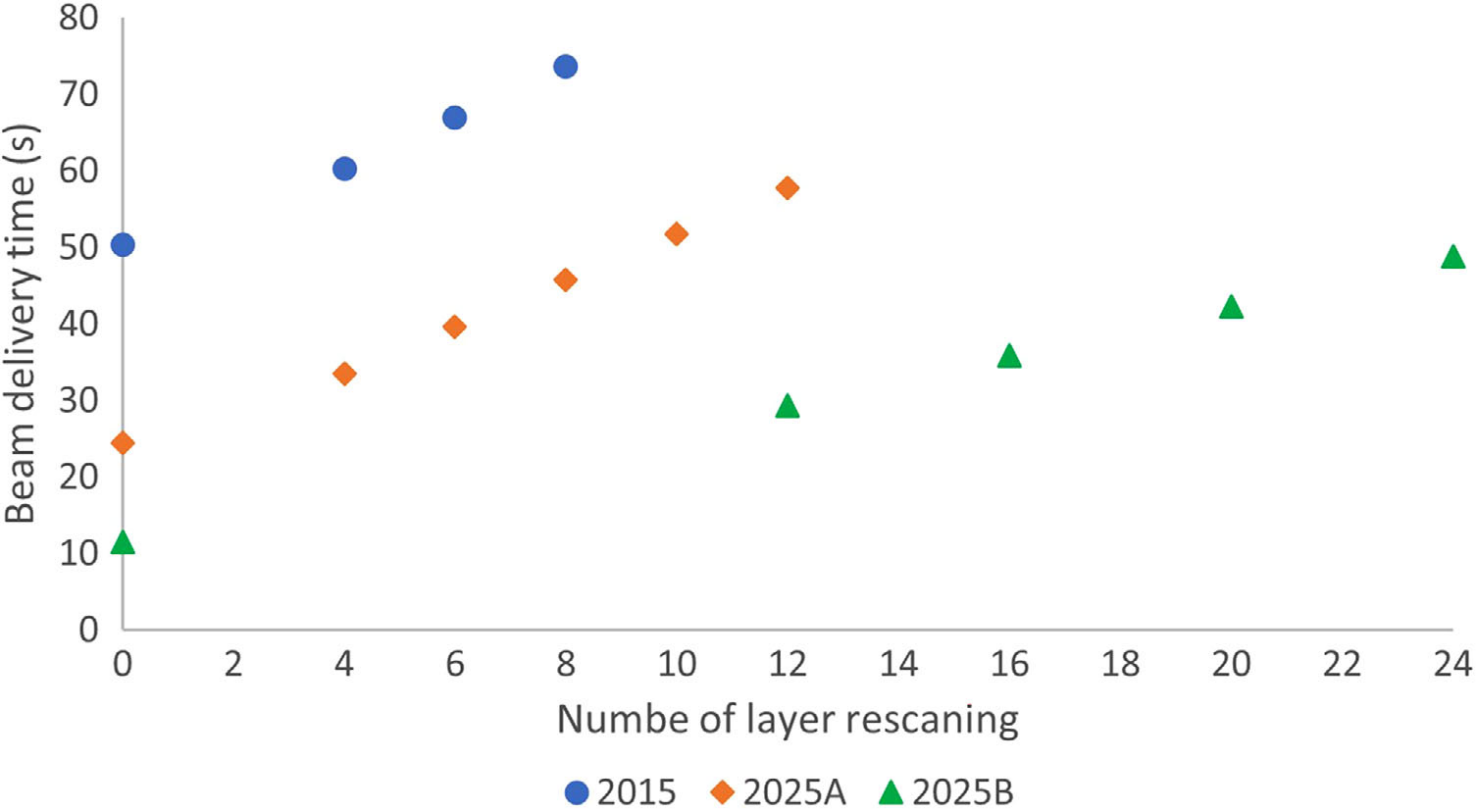
Beam delivery time for the three sets of machine operating parameters, each with various numbers of layer rescanning.

Figure 4 depicts the BDT for the three sets of machine operating parameters, each with various numbers of layer rescanning. In the absence of the rescanning, the BDTs were 50.4, 24.4, and 11.4 s for the 2015, 2025A, and 2025B machine parameters, respectively. However, the BDT increases with the number of layer rescanning. After incorporating a proper number of layer rescanning (6, 6, and 20) for interplay effect mitigation, the BDTs rose to 67.0, 39.6, and 42.3 s for the 2015, 2025A, and 2025B machine parameters, respectively. That means when using rescanning to mitigate the interplay effect, although the advanced machine operating parameters facilitates faster beam delivery, given the higher number of layer rescanning required to align the accumulated D4DD with the planned dose, the more advanced parameter machine does not necessary result in a shorter BDT.

### 3.3 Sensitivity of the respiratory phase at which the beam is initiated

**FIGURE 5:**
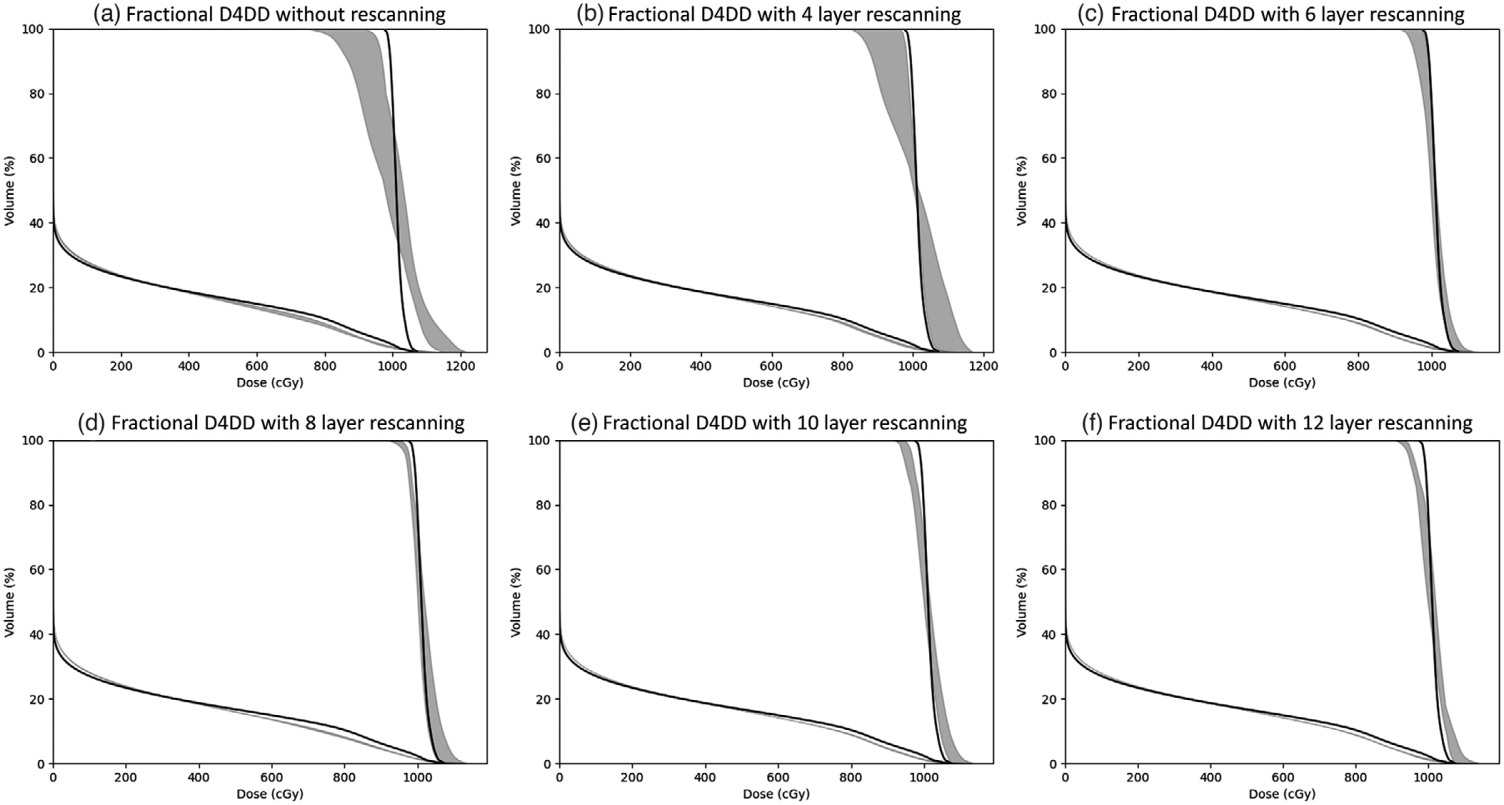
Fractional D4DD DVH band on GTV and (ipsilateral Lung—IGTV) using the 2025A machine operating parameters. (a) without rescanning, (b) with four layer rescanning, (c) with six layer rescanning, (d) with eight layer rescanning, (e) with 10 layer rescanning, and (f) with 12 layer rescanning. The gray shades depict the DVH range across the five fractions. Solid lines represent the fractional DVH of IGTV and (ipsilateral lung—IGTV) from the reference plan, serving as the reference.

To assess the influence of the respiratory phase at which beam is initiated, we evaluated fractional D4DD across the five fractions. Using the 2025A machine parameters as an example, Figure 5 illustrates the fractional D4DD DVH bands of the GTV and (ipsilateral lung—IGTV) for various numbers of layer rescanning. The range of the fractional DVH of the five fractions were showed as the gray shade. The fractional DVH furthest from the planned DVH represents the worst fraction, while the one closest to the planned DVH represents the best fraction. The fractional DVH of IGTV and ipsilateral normal lung from the reference plan is also included for comparison.

**FIGURE 6:**
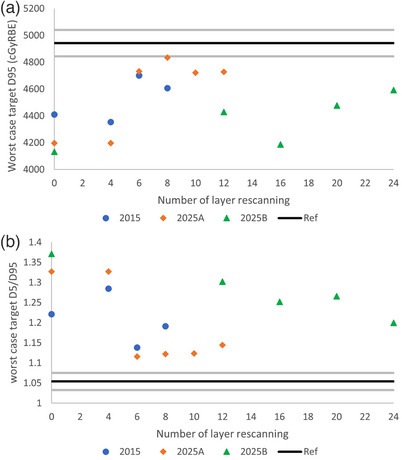
Worst‐case scenario dose for (a) GTV D95 and (b) GTV D5/D95. The black line represents the reference values on IGTV in the reference plan. The gray lines represent ±2% deviation from the reference values.

For the cumulative dose of the five fractions, the worst‐case scenario assumed that the dose is delivered in the same unfavorable manner in all fractions. Taking GTV D95 as an example, the worst‐case GTV D95 dose is calculated by multiplying the GTV D95 dose of the worst fraction by 5. Figure 6 depicted the worst‐case scenario GTV D95 and D5/D95 for the three sets of machine operating parameters, each with various numbers of layer rescanning. The reference plan values and ±2% deviations are also presented for comparison.

In a previous subsection, we illustrated that employing 6, 6, and 20 times of layer rescanning for machine parameter sets of 2015, 2025A, and 2025B, respectively, successfully aligned the accumulated D4DD with the planned dose distribution. Figures 5 and 6 demonstrated that when these specific numbers of layer rescanning were reached, the dose distribution became less influenced by at which respiratory phase the beam was initiated. The fractional D4DD DVHs across the five fractions exhibit reduced dispersion compared to those without rescanning or with fewer number of rescanning.

Using the 2025A machine as an example, another noteworthy point is that although a four times layer rescanning is not adequate for interplay effect mitigation (Figure 2), the best‐case fractional D4DD with four times layer rescanning aligned with the reference dose very well (Figure 5). Figure 6 illustrated that for the worst‐case scenario, none of the combination of rescanning and machine parameters showed the D4DD within 2% deviation from the reference plan, although Figure 2 demonstrated the interplay effect can be successfully mitigated with the proper number of layer rescanning. These observations underscore the importance of incorporating the randomness of the respiratory phase at which the beam is initiated into the evaluation of interplay effects.

## 4 DISCUSSION

The interplay effect is influenced by various factors, including beam characteristics, plan characteristic, patient characteristics, as well as machine operating characteristics. To address the specific question, we chose to perform the study using the CIRS phantom, isolating machine operating parameters as the sole variable. The tumor size of 2.5 cm, motion magnitude of 1 cm, and respiratory cycles of 5 s were chosen as they are the representative values for lung SBRT. With this controlled study design, our work provided valuable insights into how machine operating parameters impact the interplay effect and the effectiveness of rescanning in its mitigation.

This study demonstrated that more advanced machine operating parameters led to a more pronounced dose degradation due to the interplay effect. The machine with more advanced machine operating parameters requires a higher number of layer rescanning for interplay effect mitigation. Therefore, it is important to understand that, with the advancement in particle therapy machine technology, the clinical experience gained from past may not be applicable to the most up‐to‐date machines. Rescanning, by delivering spots multiple times, essentially slows down the delivery and statistically averages the spots across different breathing phases, thereby mitigating the interplay effect. To achieve effective averaging the spot delivery across respiratory phases in a fast beam delivery system, the delivery needs to be slowed down sufficiently by increasing the layer scanning numbers. This explains why a higher number of rescanning is required for advanced machine parameters.

It is worth noting that in our clinical planning practice, we set a well‐considered threshold of 3 mMU as the minimum MU constraint. For this study, we adhered to our established clinical practice, utilizing 3 mMU as the minimum MU constraint during planning, while our delivery system boasts the luxury of a minimum MU threshold as low as 0.1 mMU. Therefore, it provides ample flexibility to post‐process optimized plans for delivery using high number of layer rescanning without experiencing quality degradation due to the delivery minimum MU threshold. However, each machine operates within specific limitations, including the minimum MU per spot limitation. For delivery systems do not have such low minimum MU threshold as ours, there is a potential concern regarding plan quality when implementing high numbers of rescanning. To facilitate the delivery of plans with a high number of rescanning for each spot, such as 20 times of rescanning, the initial plan must be designed with the minimum MU per spot parameter set at least 20 times higher than the actual machine limitation. This plan parameter restriction constrains the plan optimizer's flexibility, which in turn, results in suboptimal plan quality.^30^, ^31^ In such scenarios, instead of enforcing all spots to be repainted N times, employing a technique like ‘iso‐layer scanning,^20^ or a similar approach that allows for more rescans for higher‐weighted spots than lower‐weighted spots may be a better strategy.

Our results showed when using layer rescanning to mitigate the interplay effect, despite the advanced machine operating parameters enabling faster beam delivery, given the higher number of layer rescanning required, the more advanced machine operating parameters does not necessary result in a shorter BDT. However, it is important to note that there are multiple approaches for interplay effect mitigation. For instance, if breath‐hold was the chosen method, the fast beam delivery facilitated by advanced machines, such as the 11 s in 2025B, would provide a significant advantage. Moreover, if lowering machine operating parameters leads to better plan delivery efficiency and dose accuracy, these parameters can be adjusted to optimal values. For example, with the most advanced machines such as the 2025B, if it is determined that lower than the maximum available machine operating parameters provide better dose accuracy and overall delivery efficiency, there is an opportunity to select optimal machine operating parameters on a case‐by‐case basis through a well‐designed control system. Our findings, along with the discussions mentioned above, warrant comprehensive investigations to identify the optimal number of layer rescanning and machine operating parameters for achieving high plan quality, precise dose distribution, and efficient treatment delivery.

In our study, we also made two notable observations: Firstly, although four times layer rescanning proves insufficient for interplay effect mitigation with 2025A machine parameters (Figure 2), the best‐case fractional D4DD with four times layer rescanning aligns well with the reference dose (Figure 5). This observation suggests a potential false negative with best‐case scenario. Secondly, while it is demonstrated that when the number of layer rescanning reaches a certain value, the accumulated D4DD aligns well with the reference plan dose (Figures 2 and 3), Figure 6 shows that, for the worst‐case scenario, none of the combinations of the layer rescanning number and machine parameters result in the D4DD within a 2% deviation from the reference plan. This suggests a false positive caused by the worst‐case scenario. These above‐mentioned observations emphasize the importance of considering the randomness of the respiratory phase at which the beam is initiated when evaluating the interplay effect. Failure to account for this factor may result in false negative or false positive. The randomness of the respiratory phase at which the beam starts results in the smearing of hot and cold spots across different fractions, making the accumulative D4DD acceptable when the appropriate number of rescanning is applied.

As explained earlier, our study intentionally used a phantom with regular breathing motion. However, this choice does present a limitation in our research. It is important to emphasize that patients exhibit irregular respiratory motion.^32^, ^33^, ^34^ Additionally, interplay effect is affected by beam characteristics such as spot size, planning characteristics such as spot spacing etc.^35^, ^36^, ^37^, ^38^ Therefore, our investigation does not aim to provide the guidance on the number of layer rescanning needed for each clinical setting. Instead, by varying only the machine operating parameters, our study fills the knowledge gap regarding the impact of these parameters on the interplay effect and the efficacy of rescanning in mitigating it. Comprehensive investigations that account for the aforementioned variations, associated uncertainties, as well as machine operating parameters are essential for developing institutional‐specific guidelines based on each institution's particle therapy system and treatment planning strategies. Considering the numerous factors influencing the interplay effect, such as the delivery system, treatment planning strategies, and patient characteristics, it is advisable to integrate D4DD into the workflow of plan evaluation or even plan optimization for every treatment plan involving a moving target as described by Shan et al.^39^

## 5 CONCLUSION

The effectiveness of layer rescanning for interplay effect mitigation is affected by machine operating parameters. Advanced machine operating parameters lead to a more significant dose degradation due to the interplay effect, requiring a higher number of layer rescanning. As a result, clinical experiences from the past may not be sufficiently applicable to the most up‐to‐date machines.

## AUTHOR CONTRIBUTIONS

Xiaoying Liang developed the study concept and conducted the research. Chunbo Liu created the software code. Jiajian Shen, Stella Flampouri, and Chris J. Beltran participated in in‐depth consultation discussions. Xiaoying Liang drafted the manuscript. All authors contributed clinical expertise. All authors read and approved the final version.

## CONFLICT OF INTEREST STATEMENT

The authors declare no conflicts of interest.

## Data Availability

The data that support the findings of this study are available from the corresponding author upon reasonable request.
